# Multi-institutional phase II trial of irinotecan, cisplatin, and etoposide for sensitive relapsed small-cell lung cancer

**DOI:** 10.1038/sj.bjc.6602056

**Published:** 2004-07-27

**Authors:** K Goto, I Sekine, Y Nishiwaki, R Kakinuma, K Kubota, T Matsumoto, H Ohmatsu, S Niho, T Kodama, T Shinkai, T Tamura, Y Ohe, H Kunitoh, N Yamamoto, H Nokihara, K Yoshida, T Sugiura, K Matsui, N Saijo

**Affiliations:** 1 Division of Thoracic Oncology, National Cancer Center Hospital East, 6-5-1 Kashiwanoha, Kashiwa, Chiba 277-8577, Japan; 2 Internal Medicine and Thoracic Oncology Division, National Cancer Center Hospital, Tsukiji 5-1-1, Chuo-ku, Tokyo 104-0045, Japan; 3 Department of Internal Medicine, Aichi Cancer Center Hospital, 1-1 Kanokoden, Chikusa-ku, Nagoya 464-8681, Japan; 4 Department of Internal Medicine, Osaka Prefectural Habikino Hospital, 3-7-1 Habikino, Habikino, Osaka 583-0872, Japan

**Keywords:** irinotecan, etoposide, small-cell lung cancer, sensitive relapse, second line, salvage chemotherapy

## Abstract

Irinotecan (CPT-11) has been shown to exhibit excellent antitumour activity against small-cell lung cancer (SCLC). A multi-institutional phase II study was therefore conducted to evaluate the efficacy and toxicity of CPT-11 combined with cisplatin (CDDP) and etoposide (ETOP) (PEI regimen) for the treatment of sensitive relapsed SCLC. Patients who responded to first-line chemotherapy but relapsed more than 8 weeks after the completion of first-line therapy (*n*=40) were treated using the PEI regimen, which consisted of CDDP (25 mg m^−2^) weekly for 9 weeks, ETOP (60 mg m^−2^) for 3 days on weeks 1, 3, 5, 7, and 9, and CPT-11 (90 mg m^−2^) on weeks 2, 4, 6, and 8 with granulocyte colony-stimulating factor support. Five complete responses and 26 partial responses were observed, and the overall response rate was 78% (95% confidence interval 61.5–89.2%). The median survival time was 11.8 months, and the estimated 1-year survival rate was 49%. Grade 3/4 leucocytopenia, neutropenia, and thrombocytopenia were observed in 55, 73, and 33% of the patients, respectively. Nonhaematological toxicities were mild and transient in all patients. In conclusion, the PEI regimen is considered to be highly active and well tolerated for the treatment of sensitive relapsed SCLC.

Small-cell lung cancer (SCLC) is one of the most chemosensitive solid tumours, and first-line combination chemotherapy improves survival. However, despite a high response rate to chemotherapy, the majority of SCLC patients relapse. At the time of recurrence, the tumour is broadly resistant to second-line chemotherapy and is lethal within a few to several months (Glisson, 2003). The further development of not only first-line chemotherapy but also of effective salvage chemotherapies is needed.

In predicting the efficacy of salvage chemotherapy, two major factors are important: the response to the initial chemotherapy and the duration of time between the last exposure to chemotherapy and the confirmation of recurrence (Postmus *et al*, 1987; Giaccone *et al*, 1988; Ardizzoni *et al*, 1997; Ebi *et al*, 1997). Based on these factors, relapsed SCLC is now commonly classified into two main groups. Patients who both respond to the initial chemotherapy and relapse more than 2 or 3 months after the completion of chemotherapy are considered to be ‘sensitive relapse’ patients, while patients whose tumour is stable or progresses during the initial chemotherapy or who have a recurrence within 2 or 3 months after the completion of chemotherapy are considered to be ‘refractory relapse’ patients (Giaccone *et al*, 1988). Since the outcomes of salvage chemotherapy for relapsed SCLC patients are different between these two groups, the ratios of sensitive and refractory cases must be carefully considered when evaluating the results of clinical trials for second-line chemotherapy.

The combination of cisplatin (CDDP) and etoposide (ETOP) (PE regimen) has been the standard chemotherapeutic regimen for SCLC (Fukuoka *et al*, 1991; Ihde, 1992; Roth *et al*, 1992; Aisner, 1996). Moreover, PE is a reasonable second-line chemotherapy for relapsed SCLC after combination chemotherapy consisting of cyclophosphamide, doxorubicin (ADM), and vincristine (VCR) (CAV regimen); the likelihood of a response to this regimen is 40–50% (Evans *et al*, 1984; Porter *et al*, 1985). Since PE has a relatively mild toxicity profile, other cytotoxic agent can be combined with PE.

Irinotecan (CPT-11), a camptothecin derivative topoisomerase I inhibitor, has been shown to exhibit excellent antitumour activity against SCLC in monotherapy and in combination with CDDP (Masuda *et al*, 1992; Kudoh *et al*, 1998). Based on these results, the Japan Clinical Oncology Group (JCOG) conducted a randomised phase III trial comparing CPT-11 and CDDP (IP regimen) with standard PE for previously untreated extensive stage (ED) SCLC (JCOG 9511) (Noda *et al*, 2002). The response rates were significantly higher for IP than for PE, and overall survival was also significantly better for IP than for PE. This was the first study to show the superiority of any one regimen over PE for the treatment of ED SCLC, and IP has become one of the standard regimens for ED SCLC in Japan. Thereafter, several clinical trials of CPT-11-containing regimens for patients with limited disease (LD), ED, and relapsed SCLC have been conducted by Japanese clinical study groups (Masuda *et al*, 1998; Mori *et al*, 2002; Sekine *et al*, 2002).

Consequently, a phase I trial of CPT-11 combined with weekly CDDP (25 mg m^−2^) and biweekly ETOP (60 mg m^−2^) (PEI regimen) was conducted, and the recommended dose of 90 mg m^−2^ of CPT-11 was repeated every 2 weeks (JCOG 9507) (Sekine *et al*, 2003). This regimen showed promising antitumour activity in patients with untreated ED SCLC (response rate, 91%, 1-year survival rate 46%). Moreover, since the drug dose and treatment schedule can be easily modified in a weekly regimen, this protocol is considered to be suitable for relapsed SCLC patients, who usually present with severe haematological toxicities during salvage chemotherapy because of poor bone marrow reserve (Masuda *et al*, 1990; Faylona *et al*, 1995).

Based on these results, we conducted two phase II trials to evaluate the efficacy and toxicities of PEI in patients with sensitive and refractory relapsed SCLC, separately. In this paper, the final results for the sensitive relapsed SCLC group are reported.

## PATIENTS AND METHODS

### Patient selection

Patients with histologically or cytologically confirmed SCLC who respond to first-line chemotherapy or chemoradiotherapy and relapsed more than 8 weeks after the completion of first-line treatment were candidates for the present study. Additional eligibility criteria were as follows: (1) age of 75 years or younger; (2) performance status of 0–2 on the Eastern Cooperative Oncology Group scale; (3) measurable disease; (4) adequate organ function as documented by a 4.0 × 10^9^ l^−1^⩽WBC count⩽12.0 × 10^9^ l^−1^, haemoglobin level of ⩾9.0 g dl^−1^, platelet count of ⩾100 × 10^9^ l^−1^, total serum bilirubin level of ⩽1.5 mg dl^−1^, a hepatic transaminase level of ⩽2 times the institutional upper limit of normal, a serum creatinine level of ⩽1.5 mg dl^−1^; and (5) written informed consent. Patients were not eligible for the study if they had experienced any of the following events: (1) massive pleural effusion requiring drainage; (2) prior radiotherapy with an irradiated area larger than one-third of the bone marrow volume; (3) active infection; (4) contraindications for the use of CPT-11, including diarrhoea, ileus, interstitial pulmonary fibrosis, massive ascites, or hypersensitive reaction to CPT-11; (5) serious concomitant medical illness, including severe heart disease, uncontrollable diabetes mellitus or hypertension; or (7) pregnancy or lactation. This study was approved by the institutional review board at each participating institution.

### Treatment schedule

Figure 1

**Figure 1:**
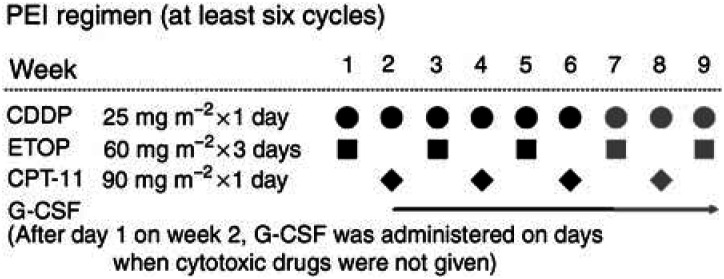
Treatment schedule.

shows the treatment schema of the PEI regimen. CDDP (25 mg m^−2^) was administered intravenously (i.v.) over 60 min on day 1 and at 1-week intervals for 9 weeks; ETOP (60 mg m^−2^) was administered i.v. over 60 min on days 1–3 of weeks 1, 3, 5, 7, and 9; and CPT-11 (90 mg m^−2^) was administered i.v. over 90 min on day 1 on weeks 2, 4, 6, and 8. Hydration (2000 ml) and granisetron (40 *μ*g kg^−1^) were given on day 1. After day 1 on week 2, granulocyte colony-stimulating factor (G-CSF) (50 *μ*g m^−2^) was administered routinely according to JCOG 9507 on days when the cytotoxic drugs were not given, unless the WBC count exceeded 10.0 × 10^9^ l^−1^. Patients were expected to complete at least six cycles of this regimen; if the toxicities were acceptable and the tumour responded to the treatment, a maximum of nine cycles of chemotherapy were performed.

### Toxicity assessment and treatment

During the course of treatment, complete blood cell counts and differential counts were analysed twice a week, and routine chemistry measurements and a chest X-ray were performed once a week. Toxicity was graded according to the toxicity criteria of the JCOG (Tobinai *et al*, 1993), a modified version of the NCI Common Toxicity Criteria issued in 1991. Grade 4 neutropenia was defined as <0.5 × 10^9^ l^−1^, and grade 3 neutropenia was defined as between (and including) 0.5–1.0 × 10^9^ l^−1^, according to the JCOG criteria. The second and subsequent cycles of chemotherapy were delayed for 1 week if one of the following toxicities was noted on day 1: a WBC count of <2.0 × 10^9^ l^−1^, a platelet count of <50 × 10^9^ l^−1^, a serum creatinine level of ⩾2.0 mg dl^−1^, an elevated hepatic transaminase level or total serum bilirubin of grade 2 or higher, diarrhoea of grades 1–2, fever ⩾38°C, or a performance status of 3. The treatment was terminated if the above-mentioned criteria did not disappear in 3 weeks or if one of the following severe nonhaematological toxicities was noted: diarrhoea of grade 2 lasting for more than 1 week, diarrhoea of grade 3, neurotoxicity of grade 3, or drug-induced pneumonitis.

### Dose modifications for toxicity

The CPT-11 dosage was reduced to 67.5 mg m^−2^ (25% reduction) in subsequent cycles if one of the following toxicities was noted: a WBC count of <1.0 × 10^9^ l^−1^, or a platelet count of <25 × 10^9^ l^−1^. If the above-mentioned toxicities reappeared after a 25% reduction in the dosage, the CPT-11 dosage was further reduced to 50 mg m^−2^ (44% reduction). Since CDDP (25 mg m^−2^) and ETOP (60 mg m^−2^) in this regimen were relatively low dose, no dose modifications for these drugs were permitted.

### Pretreatment evaluation

Pretreatment assessment included a complete blood cell count, differential counts, routine chemistry measurements, creatinine clearance, blood gas analysis, electrocardiogram, chest X-rays, computed tomography (CT) scan of the chest, brain CT scan or magnetic resonance imaging (MRI), abdominal CT scan or ultrasound sonography, radionuclide bone scan, and bone X-rays, if indicated.

### Response evaluation

Objective tumour responses were evaluated in all enrolled patients according to the WHO criteria issued in 1979 (WHO, 1979). A complete response (CR) was defined as the disappearance of all known disease for at least 4 weeks with no new lesions appearing. A partial response (PR) referred to a decrease in the total tumour size of at least 50% for at least 4 weeks without the appearance of new lesions. No change (NC) was defined as the absence of a partial or complete response and the appearance of no progressive or new lesions for at least 4 weeks. Progressive disease (PD) was defined as a 25% or greater increase in the size of any measurable lesion or the appearance of new lesions. Patients whose responses were not evaluated were included in the analysis as not evaluable (NE).

### Statistical methods

The primary end point of this study was the response rate, defined as the proportion of patients whose best response was CR or PR among all eligible patients, and its confidence interval was based on an exact binomial distribution. Simon's two-stage minimax design was used to determine the sample size and decision criteria. Assuming that a response rate of 40% in eligible patients would indicate a potential usefulness of the regimen while a rate of 20% would be the lower limit of interest and that alpha=0.05 and beta=0.20, the estimated number of required patients was 33 (Simon, 1989). Finally, this regimen would be considered worthy of further testing if 11 (33%) or more eligible patients showed an objective response. At the first stage decision, this regimen would be rejected if four (22%) or fewer of 18 eligible patients had an objective response. Thus, we determined that the sample size would be 35 registered patients. The planned accrual period was 2 years, and the follow-up period was set as 1 year after the completion of accrual. Secondary end points were toxicity and overall survival. The duration of overall survival was measured from the date of registration to the date of death from any cause or the last follow-up examination. Progression-free survival was calculated from the date of registration until evidence of PD. All patients started the treatment within 1 week of registration. The survival distribution was estimated by the method of Kaplan and Meier (1958).

## RESULTS

### Patient characteristics

From October 1998 to March 2001, 40 patients were enrolled in this study. The first-stage decision was made in October 1999, when 22 patients were registered. Three CRs and 13 PRs were observed in 18 analysed patients, resulting in a response rate of 89% (95% confidence interval (CI), 65.3–98.6%). This result did not meet the criteria for stopping the study as defined in the protocol, and the study was continued. At the time of the final analysis, there were three censored cases (8%). The median follow-up period for these cases was 25.5 months (range, 4.4–46.1 months).

The clinical characteristics of the enrolled patients are listed in Table 1

**Table 1:** Patient characteristics

Total no. of patients	40
Age, median (range)	67 (41–74)
	
*Sex*	
Male	29
Female	11
	
*ECOG performance status*	
0	9
1	30
2	1
	
*Disease extent at relapse*	
Limited disease	5
Extensive disease	35
	
*Prior chemotherapy*	
CDDP/ETOP	11
CBDCA/ETOP	11
CDDP/ETOP/CODE	6
CDDP/CPT-11	6
PEI	2
Others	4
*Prior thoracic radiotherapy*	8

ECOG=Eastern Cooperative Oncology Group; CDDP=cisplatin; ETOP=etoposide; CBDCA=carboplatin; CODE=cisplatin/vincristine/doxorubicin/etoposide; CPT-11=irinotecan; PEI=cisplatin/etoposide/irinotecan.

. Of the 40 patients in the total, 29 (73%) were male and 11 (27%) were female; the median age was 67 years. A total of 39 patients (97%) had a good performance status of 0 or 1. The extent of the disease at the time of recurrence was LD in five patients (12%) and ED in 35 (88%). All 40 patients had been previously treated using platinum-based chemotherapy, such as PE in 11 patients, carboplatin plus ETOP in 11, PE plus weekly CDDP/VCR/ADM/ETOP (CODE) in six, CDDP plus CPT-11 in six, PEI in two, and other regimens in four. Eight (20%) of these patients received thoracic radiotherapy. All patients were eligible, and the toxicity and efficacy of the regimen was evaluated in all 40 patients.

### Compliance with treatment

A total of 251 treatment cycles were administered, with a median of six cycles per patient (range, 1–9 cycles). A total of 32 patients (80%) completed six or more cycles of chemotherapy, and the median number of weeks for completing six cycles of chemotherapy was 7 weeks (range 6–10 weeks). Eight patients could not complete the planned six or more cycles for the following reasons: toxicities in four cases (grades 4 and 3 diarrhoea, grade 3 liver dysfunction, and grade 3 erythema); patient refusal in three cases; and PD in one case. Six patients (15%) had their dosage of CPT-11 reduced because of leucocytopenia in three, thrombocytopenia in two, and both in one.

### Clinical response and survival

**Figure 2:**
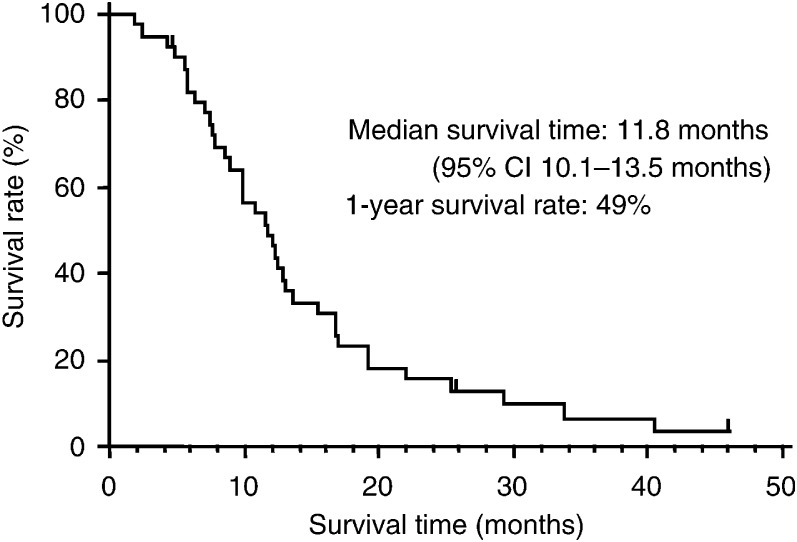
Overall survival (*n*=40).

All the patients were included in the analyses of tumour response and survival. Five CRs (13%) and 26 PRs (65%) were observed, for an overall response rate of 78% (31 out of 40 patients; 95% CI, 61.5–89.2%). Four NC, four PD, and one NE were also observed. One patient was lost to follow-up and only two patients were still alive as of April 16, 2003. The median survival time (MST) was 11.8 months (95% CI, 10.1–13.5 months), and the estimated 1-year survival rate was 49% (Figure 2

).

### Site of first relapse and progression-free survival

**Figure 3:**
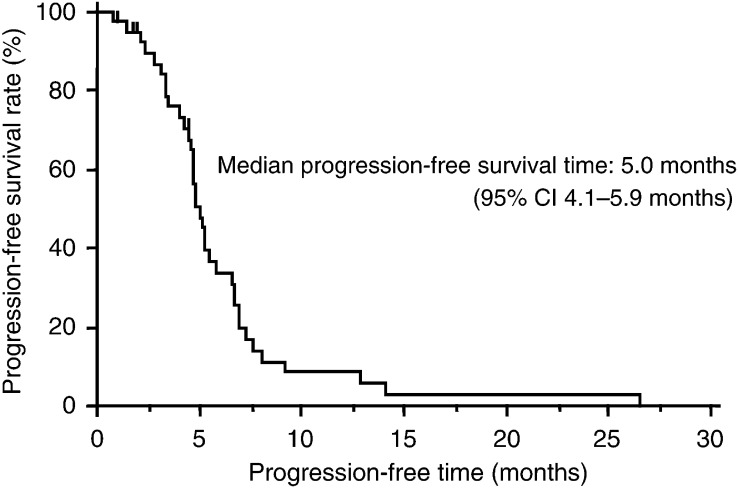
Progression-free survival (*n*=40).

The majority of patients (*n*=30, 75%) experienced a systemic relapse after completing PEI, including 17 patients (43%) with central nerve metastases. Six patients (15%) developed only a locoregional recurrence, and one had no recurrence and died of acute myocardial infarction. No data on recurrence patterns were available in three patients because these patients were followed up at other hospitals. In all, 13 patients received additional chemotherapy treatment after recurrence (no data on response to third-line chemotherapy were available), while four patients underwent palliative chest radiotherapy and 18 underwent whole-brain irradiation for cerebral metastases. One patient, who achieved a CR by this regimen, developed a locoregional recurrence and underwent a right upper lobectomy. He has not experienced any further relapse and is still alive. The median progression-free survival period was 5.0 months (95% CI, 4.1–5.9 months) (Figure 3

).

### Toxicities

All the patients were included in the toxicity analysis. Severe toxicities were mainly haematological. Grades 3–4 leucopenia, neutropenia, and thrombocytopenia were observed in 22 (55%), 29 (73%), and 13 (33%) patients, respectively (Table 2

**Table 2:** Haematological toxicities (JCOG toxicity criteria)

	**0**	**1**	**2**	**3**	**4**	**% of Grs 3 and 4**
Leucocytopenia	2	3	13	17	5	55
Neutropenia	3	4	4	12	17	73
Anemia	2	4	16	18	—	45
Thrombocytopenia	10	7	10	7	6	33
Elevated total bilirubin	33	—	6	1	0	3
Elevated GOT	32	7	0	1	0	3
Elevated GPT	30	7	2	1	0	3
Elevated creatinine	37	3	0	0	0	0
Hyponatremia	28	4	6	0	2	5
Hypokalemia	32	5	3	0	0	0

Grs=grades; GOT=glutamic oxaloacetic transaminase; GPT=glutamic pyruvic transaminase.

). Nonhaematological toxicities were mild and transient in all patients. Grades 3–4 diarrhoea was noted in only three patients (8%) (Table 3

**Table 3:** Nonhaematological toxicities (JCOG toxicity criteria)

	**0**	**1**	**2**	**3**	**4**	**% of Grs 3 and 4**
PS	1	30	4	5	0	13
Infection	28	4	7	1	0	3
Fever	29	7	4	0	0	0
Nausea/vomiting	11	15	11	3	—	8
Diarrhoea	15	16	6	2	1	8
Mucositis	36	4	0	0	0	0
Arrythmia	36	2	0	1	1	5
Eruption	37	1	1	1	0	3
Alopecia	16	17	7	—	—	—
Allergy	39	0	1	0	0	0

Grs=grades; PS=performance status.

). No treatment-related deaths occurred.

## DISCUSSION

Despite a high response rate to first-line chemotherapy, most patients with SCLC experience a relapse within a year of the completion of therapy (Hansen, 1992). Although many relapsed patients in good physical condition undergo second-line chemotherapy, the results are disappointing. The obtained response is usually brief, and the median survival period is generally less than 4 months (Albain *et al*, 1993; Glisson, 2003).

Although one phase III trial for patients with relapse SCLC comparing the use of topotecan with CAV has been reported (von Pawel *et al*, 1999), a standard treatment for relapsed SCLC has not been agreed upon. However, the repeated use of the original induction regimen is the most popular treatment for sensitive relapsed patients. Reinduction chemotherapy has been reported to produce a response rate of 50%, and patients who relapsed more than 3 months after the end of their previous chemotherapy regimen were sensitive to reinduction chemotherapy (Giaccone *et al*, 1987; Postmus *et al*, 1987). Giaccone *et al* (1988) suggested that sensitive tumour cells, which were not completely eradicated by the induction chemotherapy, regrow spontaneously after the suspension of chemotherapy, eventually constituting a clinically significant part of the tumour burden. In the present study, two patients received the PEI regimen as a reinduction chemotherapy, and both patients showed PRs.

Many clinical trials of salvage chemotherapy for relapsed SCLC have been reported. In these studies, the single administration of CPT-11 or ETOP produced good results, with response rates of 16–47% and an MST of 3.5–6.2 months (Einhorn *et al*, 1990; Johnson *et al*, 1990; Masuda *et al*, 1992; Le Chevalier *et al*, 1997). Moreover, CPT-11 or ETOP-containing combined chemotherapy regimens showed favourable results, with response rates of 20–88% and an MST of 4.7–8.7 months (Table 4

**Table 4:** Combination chemotherapy studies for relapsed small-cell lung cancer

**Author**	**Regimen**	**No. of pts**	**% of ref pts (%)**	**RR (%)**	**RR in ref pts (%)**	**MST (month)**
Sculier	CAV	61	75	21	5	6.2–7.5
von Pawel	CAV	104	20	18	5	6.2
Roth	CAV	41	32	12	8	NM
Roth	PE	59	46	22	15	NM
Evans	PE	78	50	55	28	NM
Masuda	PE	20	NM	50	NM	4.7
Gridelli	CCNU/MTX	33	100	21	21	4.0
Faylona	PE/IFO	46	41	55	50	6.8
Kubota	CODE	17	35	88	83	8.2
Masuda	CPT-11/ETOP	25	16	71	75	8.7
Nakanishi	CPT-11/CDDP	5	100	20	20	NM
Domine	GEM/PTX	31	58	50	40	NM
Groen	CBDCA/PTX	35	100	74	74	7.2
Kosmas	CDDP/IFO/PTX	33	61	73	70	6.5

Pts=patients; ref=refractory; RR=response rate; MST=median survival time; CAV=cyclophosphamide/doxorubicin/vincristine; PE=cisplatin/etoposide; CCNU= lomustine; MTX=methotrexate; IFO=ifosfamide; CODE=cisplatin/vincristine/doxorubicin/etoposide; CPT-11=irinotecan; ETOP=etoposide; CDDP=cisplatin; GEM=gemcitabine; PTX=paclitaxel; CBDCA=carboplatin; NM=not mentioned.

) (Evans *et al*, 1985; Masuda *et al*, 1990; Sculier *et al*, 1990; Gridelli *et al*, 1991; Roth *et al*, 1992; Faylona *et al*, 1995; Kubota *et al*, 1997; Masuda *et al*, 1998; Groen *et al*, 1999; Nakanishi *et al*, 1999; von Pawel *et al*, 1999; Domine *et al*, 2001; Kosmas *et al*, 2001). Therefore, these two drugs are considered to be key drugs for the treatment of relapsed SCLC. In particular, the combination of CPT-11 and ETOP (a combination of topoisomerase I and II inhibitors) produced a high response rate (71%) and the best survival results (MST, 8.7 months) (Masuda *et al*, 1998). In addition, a weekly chemotherapy regimen containing ETOP (CODE) was highly active in patients with relapsed SCLC, with a favourable response rate (88%) and survival duration (MST, 8.2 months) (Kubota *et al*, 1997). In the two studies mentioned above, four patients (16%) with refractory relapsed SCLC were included in the CPT-11 and ETOP study, and six patients (35%) with refractory relapsed SCLC were included in the CODE study. Three and five of these patients achieved PR, respectively.

The response and survival data from Japanese clinical trials for relapsed SCLC were generally better than those obtained in western countries. We have no proof that this difference depends on either drug metabolism or tumour sensitivity. It is possibly related to the difference in patient follow-up interval between Japan and western countries. Since intensive follow up after completion of first-line treatment is common in Japan, relapses can be detected in the early stage by CT or MRI before becoming symptomatic. Therefore, relapsed patients had a relatively good performance status, and showed good responses to second-line chemotherapy as well as better survival results.

The weekly regimen was designed to increase the overall relative dose intensity of the chemotherapeutic drugs (Murray *et al*, 1991). However, several phase III trials have made it clear that intensive weekly chemotherapy does not improve the survival of patients with SCLC (Furuse *et al*, 1998; Murray *et al*, 1999). On the other hand, drug dosages and treatment schedules are easy to modify in weekly chemotherapy regimens. Since patients with relapsed SCLC may have lower bone marrow reserve, a high-dose regimen or intensified dosage can lead to treatment-related death (Masuda *et al*, 1990; Faylona *et al*, 1995). In the PEI regimen, the individual dosage of each drug is within the commonly used range and the dose given at one time is lower than that of a standard 3-week cycle regimen. The PEI regimen therefore permits greater flexibility in dosage adjustment and treatment delays based on laboratory data or the physical condition of patients. Thus, this regimen is considered to be suitable for the treatment of patients with relapse SCLC. In addition, this weekly schedule may be of great advantage for enabling the synergistic effects of ETOP (a topoisomerase II inhibitor) and CPT-11 to be realised because the development of resistance to topoisomerase II inhibitors has been reported to increase tumour sensitivity to subsequent treatment with topoisomerase I inhibitors (Vasey and Kaye, 1997).

Three cytotoxic drugs were used in this PEI regimen. However, three-drug combination chemotherapy was reportedly associated with more severe toxicity and showed no survival benefit as compared with the two-dug combination (Mavroudis *et al*, 2001; Niell *et al*, 2002). The main reason for mild toxicities was that the PEI regimen consists of a weekly schedule. With a weekly chemotherapy regimen, drug dosages and treatment schedules can easily be adjusted according to haematological data and the patient's physical condition. These careful modifications resulted in a mild toxicity profile with the PEI regimen. Moreover, the PEI regimen did not consist of concomitant administration of three drugs but rather weekly alternative administration of a two-drug combination chemotherapy, that is, PE and IP. As a result, the toxicity profile was similar with that of two-drug combination chemotherapy.

Although all the patients in this study were sensitive relapsed cases, the overall response rate of 78% is one of the best results reported for relapsed SCLC. Moreover, although only selected patients with a good performance status were included in this study, it is notable that the median survival time was 11.8 months and the 1-year survival rate was 49%. In JCOG- 9511, the MST was 12.8 months in the IP arm and 9.4 months in the PE arm for chemotherapy naive ED SCLC patients (Noda *et al*, 2002). Our survival data for PEI is almost equivalent to that of first-line treatment. Salvage chemotherapy may be possible to prolong the survival of sensitive relapsed SCLC patients who are in good physical condition.

Since second-line chemotherapy for relapsed SCLC patients is a palliative treatment, a reasonable toxicity profile is essential. The main toxicities of the PEI regimen were haematological. Although G-CSF was routinely administered, Grades 3–4 leucopenia and neutropenia were observed in 55 and 73% of patients, respectively. Grades 3–4 thrombocytopenia was observed in 33% of patients. However, the frequencies of these haematological toxicities were approximately equal to that of first-line PE treatment (Noda *et al*, 2002). Nonhaematological toxicities were mild and transient in all patients. Grades 3–4 diarrhoea was noted in only three patients (8%). Irinotecan dose modifications as a result of haematological toxicities were only performed in six patients (15%). All toxicities were easily manageable, and no treatment-related deaths occurred.

In conclusion, PEI is a highly active and well-tolerated treatment for sensitive relapsed SCLC. Another phase II trial restricted to refractory relapsed SCLC patients is presently being performed by our clinical group. Further phase III studies comparing PEI regimen with rechallenges of the same drugs used in the first-line chemotherapy regimen should clarify the role of second-line chemotherapy for sensitive relapsed SCLC and are now being planned.
